# Transcriptomic Analysis Identifies RNA Binding Proteins as Putative Regulators of Myelopoiesis and Leukemia

**DOI:** 10.3389/fonc.2019.00692

**Published:** 2019-08-06

**Authors:** Subha Saha, Krushna Chandra Murmu, Mayukh Biswas, Sohini Chakraborty, Jhinuk Basu, Swati Madhulika, Srinivasa Prasad Kolapalli, Santosh Chauhan, Amitava Sengupta, Punit Prasad

**Affiliations:** ^1^Epigenetic and Chromatin Biology Unit, Institute of Life Sciences, Bhubaneswar, India; ^2^Translational Research Unit of Excellence (TRUE), Stem Cell and Leukemia Laboratory, Council of Scientific and Industrial Research (CSIR)-Indian Institute of Chemical Biology (IICB), Kolkata, India; ^3^Department of Pathology, New York University School of Medicine, New York, NY, United States; ^4^Cell Biology and Infectious Disease Unit, Institute of Life Sciences, Bhubaneswar, India

**Keywords:** RNA binding proteins (RBP), myeloid development, acute myeloid leukemia (AML), hematopoietic stem cells, leukemic stem cells

## Abstract

Acute myeloid leukemia (AML) is a common and aggressive hematological malignancy. Acquisition of heterogeneous genetic aberrations and epigenetic dysregulation lead to the transformation of hematopoietic stem cells (HSC) into leukemic stem cells (LSC), which subsequently gives rise to immature blast cells and a leukemic phenotype. LSCs are responsible for disease relapse as current chemotherapeutic regimens are not able to completely eradicate these cellular sub-populations. Therefore, it is critical to improve upon the existing knowledge of LSC specific markers, which would allow for specific targeting of these cells more effectively allowing for their sustained eradication from the cellular milieu. Although significant milestones in decoding the aberrant transcriptional network of various cancers, including leukemia, have been achieved, studies on the involvement of post-transcriptional gene regulation (PTGR) in disease progression are beginning to unfold. RNA binding proteins (RBPs) are key players in mediating PTGR and they regulate the intracellular fate of individual transcripts, from their biogenesis to RNA metabolism, via interactions with RNA binding domains (RBDs). In this study, we have used an integrative approach to systematically profile RBP expression and identify key regulatory RBPs involved in normal myeloid development and AML. We have analyzed RNA-seq datasets (GSE74246) of HSCs, common myeloid progenitors (CMPs), granulocyte-macrophage progenitors (GMPs), monocytes, LSCs, and blasts. We observed that normal and leukemic cells can be distinguished on the basis of RBP expression, which is indicative of their ability to define cellular identity, similar to transcription factors. We identified that distinctly co-expressing modules of RBPs and their subclasses were enriched in hematopoietic stem/progenitor (HSPCs) and differentiated monocytes. We detected expression of DZIP3, an E3 ubiquitin ligase, in HSPCs, knockdown of which promotes monocytic differentiation in cell line model. We identified co-expression modules of RBP genes in LSCs and among these, distinct modules of RBP genes with high and low expression. The expression of several AML-specific RBPs were also validated by quantitative polymerase chain reaction. Network analysis identified densely connected hubs of ribosomal RBP genes (rRBPs) with low expression in LSCs, suggesting the dependency of LSCs on altered ribosome dynamics. In conclusion, our systematic analysis elucidates the RBP transcriptomic landscape in normal and malignant myelopoiesis, and highlights the functional consequences that may result from perturbation of RBP gene expression in these cellular landscapes.

## Introduction

Acute myeloid leukemia (AML) is characterized by uncontrolled proliferation of immature blast cells and is one of the most common forms of leukemia in adults. The blast cells arise from leukemic stem cells (LSCs), which are derived from hematopoietic stem cells (HSCs) that have accumulated genetic mutations and epigenetic aberrations. Current chemotherapeutic treatment includes drugs such as cytarabine and anthracycline, which target the hyper-proliferative blasts but spare the LSCs, thereby causing AML relapse with poor survival rates. The transcriptional and epigenetic mechanisms underlying the onset and progression of different cancers including AML are being extensively evaluated (1, 2). Post-transcriptional gene regulation (PTGR), which shapes the transcriptome per normal differentiation cues and simultaneously prevents aberrant gene expression has not been extensively studied in the context of normal and malignant hematopoiesis. RNA binding proteins (RBPs), which form ribonucleoprotein complexes (RNPs) are the central players in PTGR and are involved in normal development, perturbation of which results in various cancers, including AML (3). Recently, interest in investigating the role of RBPs as regulators of PTGR in normal myeloid differentiation and their dysregulation in AML pathogenesisis has increased. RNA metabolism, including RNA processing, transportation, modification, and degradation, is largely coordinated by the conserved RNA binding domains (RBDs) of the RBPs. Despite the general role of RBPs in RNA biogenesis and processing, recent findings suggest that a subset of RBPs are also expressed in tissue-specific manner and are possibly involved in cell fate decisions (3).

Pan-cancer analysis has revealed common driver mutations, somatic copy number alterations (SCNA), and altered mRNA expression of RBPs in various cancers including AML (4). Neelamraju et al. has reported that 50% of RBPs and transcription factors (TFs) are mutated in certain cancers. Furthermore, the mutated RBPs are involved in cellular pathways such as translation, splicing and apoptosis (4). Wang et al. has reported cancer-type-specific driver mutations and SCNAs in RBPs (5). Mutations in various RBPs splicing factors such as SRSF2, SF3B1 and U2AF1 have been reported to contribute to myelodysplasia, AML and other blood-related disorders owing to altered binding capacity and dysregulated splicing events (6–9). Mutations in the ribosomal RBP, RPS14, contribute to myelodysplasia owing to defective 18S rRNA processing (10). CRISPR/Cas9-mediated screening of 490 RBPs in AML cell lines revealed 21 RBP candidates that are upregulated in AML and are essential for AML cell survival (11).

In the present study, we have systematically utilized high-throughput transcriptomic data to identify RBPs that are potential regulators of normal myeloid development and leukemia. Toward this, we have collated a list of 1,734 RBPs and analyzed their expression in hematopoietic stem cells (HSCs), common myeloid progenitors (CMPs), granulocyte-macrophage progenitors (GMPs), and monocytes during normal myeloid development, and in leukemic stem cells (LSCs) and blasts during leukemic cell development. We have identified distinct, as well as co-expressing RBPs in the hematopoietic stem/progenitor (HSPCs) and in differentiated monocytes. We further identified a set of RBPs that were specifically associated with LSCs, and network analysis revealed densely interconnected ribosomal RBP (rRBP) gene hubs with significantly lesser expression in LSCs than in HSCs/blasts. This observation suggests the dependency of LSCs on altered ribosome dynamics to maintain a cancer-specific translatome. We also experimentally validated the expression of a RBP gene, DZIP3, in a cell line and showed that loss of DZIP3 enhances monocytic differentiation. We also validated the expression of four important AML specific RBP genes, *CLK4, ERI1, NSUN7* and *RBM47*. In conclusion, our study aims to provide a comprehensive picture of the expression and function of RBPs in myelopoiesis and leukemic transformation and highlights their importance as potential candidates for therapeutic intervention toward effective eradication of LSCs in AML.

## Materials and Methods

### RNA-Sequencing Data Curation and Processing

A comprehensive list of 1,734 RBPs for this study was curated from Gerstberger et al. (3) and Bhargava et al. (12) (Supplementary Table 1). Gene expression data for HSCs (*n* = 4), CMPs (*n* = 4), GMPs (*n* = 4), monocytes (*n* = 4), LSCs (*n* = 8), and blasts (*n* = 11) were downloaded from the Gene Expression Omnibus (GEO), from the dataset GSE74246 (https://www.ncbi.nlm.nih.gov/geo/query/acc.cgi?acc=GSE74246) using NCBI sratoolkit (v2.8.2-1) (13). The “.sra” files were converted to fastq format using the “fastq-dump” function from sratoolkit. Quality checks were run using FastQC (v0.11.5) (www.bioinformatics.babraham.ac.uk/projects/fastqc/), followed by adapter trimming using BBDuk (v37.58). Sequence alignment was performed using STAR aligner (v2.5.3a), with default parameters, and Gencode v 21, GRCh38) (14), was used as the genome reference for annotation purposes. Post-alignment, duplicates were removed using Picard (v2.9.4) and the “bam” files were indexed using samtools (v1.4.1). To generate a count matrix for each comparison, “featureCounts” (v1.5.3) from the subread-1.5.3 package was used, with *Q* = 10 for mapping quality. These count files were used as input for differential gene expression analysis with DESeq2 (v1.14.1) (15). Read counts < 10 in all the samples were first removed and the remaining data were regularized log (rlog) transformed Statistical significance was calculated using default parameters, and genes were selected based on log_2_ fold change greater/less than 1.5 and adjusted *p* ≤ 0.05. We have compared the RBP gene expression profile of HSCs with those of CMPs, GMPs and monocytes (normal myelopoiesis) and those of LSCs with blast for AML samples.

### Analysis of Gene Expression Profiles

Principal component analysis (PCA) was performed using the base R function “prcomp.” The first three principal components explaining more than 50% variance were plotted using the “scatterplot3d” (v0.3.41) package. Spearman correlation matrix between cell types was calculated using the base “Rcor” function. The “corrplot” (v0.84) package was used for clustering and visualization. Pairwise correlation between genes was calculated using the “Hmisc” (v4.1-1) package, and the results were used as input for data clustering and visualization, which was done using “pheatmap” (v1.0.10). The heat map for unsupervised hierarchical clustering was plotted using “ComplexHeatmap” (v1.18.1) package where the expression matrix was transformed into *z*-score. UpSet plots were generated using “UpSetR” R-package for generating intersections of RBP sets and sizes between different modules and RBPs listed in different databases (16).

### Shortest Path Analysis

The undirected human protein-protein interaction (PPI) was downloaded from the STRING database (v10.0) (17) and only interactions with confidence score ≥ 500 were retained. Differentially regulated genes (DRGs) were annotated with Ensembl protein IDs (Ensembl Gene74) and assigned with unique index IDs using custom Perl scripts. Using the R “igraph” package, all possible shortest paths (SPs) of gene interactions between the RBP genes [one RBP gene acts as source node and another RBP gene acts as target node, with intermediate nodes (genes) in a SP being either an RBP gene or a non-RBP gene] were obtained. Subsequently, SPs were selected in which each of the nodes (i.e., genes) in the path showed statistical significance (adj. *p* ≤ 0.05) in the difference in expression between LSCs and HSCs. Networks of gene interaction from these SPs were created and the degree of interaction of each node (gene) with other nodes in the network was analyzed. Genes with the top 10 percentile of degree values were identified as “hub” genes within each network.

### Cell Culture and Transduction

Human leukemia cell line (HL60) CCL-240™ and U937 cells CRL-1593.2™ were obtained from the American Type Culture Collection (ATCC)® and cultured as per ATCC's recommendations. The cell density was maintained at 0.3–0.8 × 10^6^ cells/ml. HEK293T cells were cultured in Dulbecco's modified Eagle's medium (DMEM) (Gibco, 31966047) media supplemented with 10% FBS (Gibco, 10270106) and 1% Penicillin Streptomycin (Gibco, 15140122). Cells were seeded at density of 0.3 × 10^6^ cells/ml and were spilt upon reaching 80% confluence.

Lentiviral preparation was made in HEK293T cells after transfection with the pLKO transfer plasmid bearing shRNA sequences or the empty vector (Sigma) and packaging plasmids (pMDG.2 and pCMVR) using a modified version of the Cal-Phos (CaPO_4_) mammalian transfection kit (Clontech, 631312) protocol and the method used by Salmon and Trono (18). Briefly, the culture media was changed after 12 h, and the supernatant containing the virus was collected after 24 h, followed by membrane filtration using 0.45 μm filter, aliquoting in smaller volumes, and storage at −80°C until further use. The U937 cells (0.6 × 10^6^ cells/ml) were transduced with the viral supernatant overnight in the presence of polybrene (Sigma, TR-1003), followed by media change. After 48 h, the cells were subjected to puromycin (Sigma, P8833) selection for another 48 h and harvested for protein/RNA or differentiation assays.

### Differentiation Assay

HL60 and U937 cells (wild type or transduced) were treated with 50 nM and 30 nM of 1α,25-dihydroxyvitamin D3 (Sigma, D1530), respectively for 72 h to differentiate into monocytes, which was assessed by scoring CD14+ cells (BD Biosciences, 560180) cells using CytoFlex S (Beckman Coulter). Data was analyzed using CytExpert (Beckman Coulter).

### Western Blotting

The crude lysate of U937 cells was prepared by disrupting the cells via sonication (10 cycles) using a picobioruptor (Diagenode) in RIPA buffer (140 mM NaCl, 1 mM EDTA, 1% Triton X-100, 0.1% SDS, 0.1% sodium deoxycholate, 10 mM Tris-Cl pH 8.0, supplemented with 2 X Roche protease inhibitor cocktail, 1 mM PMSF, 2X neutrophil elastase inhibitor (Sigma, M0398). Approximately, 70 μg protein was run in 4–12% Bis-Tris gradient gel (Invitrogen, NP0322BOX), followed by semi-dry transfer to polyvinylidene fluoride (PVDF) membrane (Bio-Rad Transblot). Western blotting was performed using anti-DZIP3 (Sigma, SAB2701600, 1:2,000) and anti-actin (GeneTex, GTX26276, 1:5,000) antibodies. Horse radish peroxidase (HRP)-conjugated anti-rabbit IgG and IR800-conjugated anti-mouse IgG were used as secondary antibodies and the blot was visualized using a Chemidoc system (Bio-Rad).

### RNA Isolation and RT-qPCR

For cell lines, total RNA was isolated using a kit from Zymo Research (R2052) per the manufacturer's protocol. The quality and quantity of RNA were assessed using a spectrophotometer. RNA was converted to cDNA using a cDNA synthesis kit from Thermo Scientific (Maxima first strand cDNA synthesis kit for RT-qPCR; K1672) following the manufacturer's protocol. RT-qPCR was performed using SYBR mix (ABI, 4368577) in Quant6 Studio from ABI Biosystems. The DZIP3 Ct values were normalized to that of *ACTB1* as an internal control. The relative fold change of DZIP3 over the vector control was calculated using the 2^−ΔΔ*Ct*^ method.

For samples from AML patients, total RNA was isolated using TRIzol (Life Technologies) according to the manufacturer's recommendation. Genomic DNA contamination was removed using RNase-free DNase I recombinant kit (Roche, 4716728001). RNA amount was quantified and cDNA was prepared using TaqMan reverse transcription reagents (Applied Biosystems, 8080234). Gene expression levels were determined using quantitative PCR with SYBR Select master mix (Applied Biosystems, 4472908) on a 7500 Fast real-time PCR system (Applied Biosystems). The reverse transcribed cDNA was used as the template. GAPDH was used as a housekeeping gene. Relative expression levels were calculated using the 2^−ΔΔ*Ct*^ method. Supplementary Table 10 shows the list of primers used in this study.

### Patient Cohort

Human (*n* = 28) bone marrow (BM) aspirates (1–2 ml each) were obtained from Park Clinic, Kolkata, India, from newly diagnosed, untreated patients. Informed consent was obtained from all patients. The study protocol was approved by the Institutional Human Ethics Committee and following the guidelines set by the Council of Scientific and Industrial Research–Indian Institute of Chemical Biology (CSIR-IICB) Institutional Review Board. Sample collection was part of routine diagnosis, and the inclusion criterion for this study was histopathological confirmation of bone marrow aspirates or biopsies and immunophenotypic analyses as reported earlier (19–21). BM aspirates were also collected from age-matched normal individuals (*n* = 7) who were pathologically negative for AML after obtaining informed consent. Low density (1.077 gm/cc) nuclear cells from AML BM or normal BM samples were isolated using Ficoll (Sigma) separation and cryopreserved in liquid nitrogen.

## Results

### RBPs in Myeloid Development

**Figure 1:**
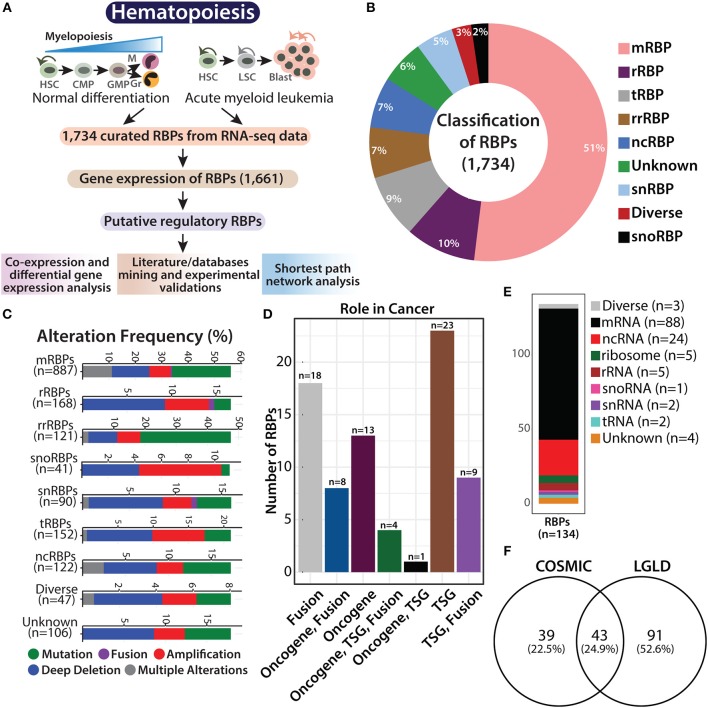
Overview of RNA binding proteins (RBPs). Flowchart showing hematopoietic hierarchy during normal differentiation and leukemia, followed by the systematic work plan adapted in this study to understand the potential role of RBPs in normal and malignant hematopoiesis **(A)**. Pie graph showing the classification of 1,734 RBPs based on their function and RNA counterparts, such as mRNA binding (mRBP), ribosome (rRBP), tRNA binding (tRBP), rRNA binding (rrRBP), ncRNA binding (ncRBP), snRNA binding (snRBP), and “diverse” targets, and RBPs with “unknown” interacting partner **(B)**. Stacked bar plots showing genetic alteration frequency of RBP functional classes in AML TCGA dataset comprising of 162 patients **(C)**. Bar plot showing distribution of 82 RBPs across different cancers identified from Cosmic database; TSG (tumor suppressor gene) **(D)**. Stacked bar plot depicting RBP subclasses reported in LGL database **(E)**. Venn diagram showing overlap between RBP genes identified from COSMIC database and LGL database **(F)**.

As key players of PTGR, RBPs modulate the fate of transcripts and regulates translational output in multiple ways. The landscape of RBP expression across different solid tumors has been dissected, which shows their involvement in cancer pathogenesis (4, 22). Tuschl's group showed that the co-expression patterns of RBPs change during fetal ovarian and hippocampal development, suggesting that the PTGR by RBPs are crucial during development However, similar studies have not been performed for hematopoietic development and myeloid neoplasms such as AML. To understand the role of RBPs in normal and malignant myelopoiesis, we have compiled a list of 1,734 RBPs from two studies described in the Methods, where the authors have classified RBPs as bona fide RBPs, based on their domain architecture, evolutionary significance, type of RNA-RBP interaction and their functions (3, 12). The workflow schema for gene expression analysis of RBPs in normal and malignant myelopoiesis is summarized in Figure 1A. Briefly, the myeloid lineage of human hematopoiesis originates from HSCs and differentiates into CMP and GMP progenitor populations, which give rise to mature monocytes and granulocytes. However, in AML, owing to a differentiation block, leukemic blast cells derived from LSCs do not differentiate further and accumulate within the bone marrow. The expression profile of the curated list of 1,734 RBPs was extracted from the high-throughput transcriptome data of GSE74246 (https://www.ncbi.nlm.nih.gov/geo/query/acc.cgi?acc=GSE74246) for the above-mentioned cell types (13). Out of the starting set of 1,734 RBPs, 1,661 RBPs were detected in our analysis of the RNA-sequencing data (Supplementary Table 2), which were subsequently used for downstream analysis in normal and malignant myeloid cells (Figure 1A).

RBPs regulate transcript levels within cells in multiple ways and perform various functions related to RNA metabolism. As described by Gerstberger et al., the curated RBPs were subclassified as mRNA-binding proteins (mRBPs), ribosomal proteins (rRBPs), pre-rRNA-binding proteins (rrRBPs), tRNA-binding proteins (tRBPs), small nuclear RNA (snRNA)-binding proteins (snRBPs), small nucleolar RNA (snoRNA)-binding proteins (snRBPs) and non-coding RNA-binding proteins (ncRBPs), RBPs with more than one type of RNA as an interacting partner are categorized as “diverse” RBPs, while RBPs that harbor more than one conserved RBD, the interacting RNA partner of which in the human RNA-protein interactome is not known are categorized as “unknown” [(3, 12); Figure 1B].

Recent studies have shown that somatic copy number alterations (SCNA), mutations, and alterations in the expression of RBPs potentially contribute to tumorigenesis (4, 5). We attempted to determine the abundance of genetic aberrations in genes encoding RBPs in AML patients. We retrieved data on genetic alterations associated with the 1,734 RBPs, in 162 patients with AML, from cBioPortal for Cancer Genomics database (https://www.cbioportal.org/) [(23, 24); Figure 1C]. Deep deletions were the most common form of genomic alteration in all RBP classes; snoRBP genes, however showed the highest number of gene amplification. We also curated information regarding gene mutations in these RBP genes from COSMIC database (https://cancer.sanger.ac.uk/cosmic), and observed that 82 of these RBP genes possess mutations that drive the cancer phenotype (Figure 1D and Supplementary Table 3). We compared our list of RBPs with the genes reported to be involved in AML with the LGL database (http://soft.bioinfo-minzhao.org/lgl/) and identified 134 common genes distributed across various sub-classes of RBPs (Figure 1E). Next, we detected an overlap of 43 RBP genes that are reported to be frequently mutated in cancer between the COSMIC database and the RBP genes involved in leukemia identified via literature mining in the LGL database (Figure 1F). This analysis confirms that the RBP genes are frequently altered in patients with AML and other cancers.

### Expression of RBPs During Myeloid Differentiation

**Figure 2:**
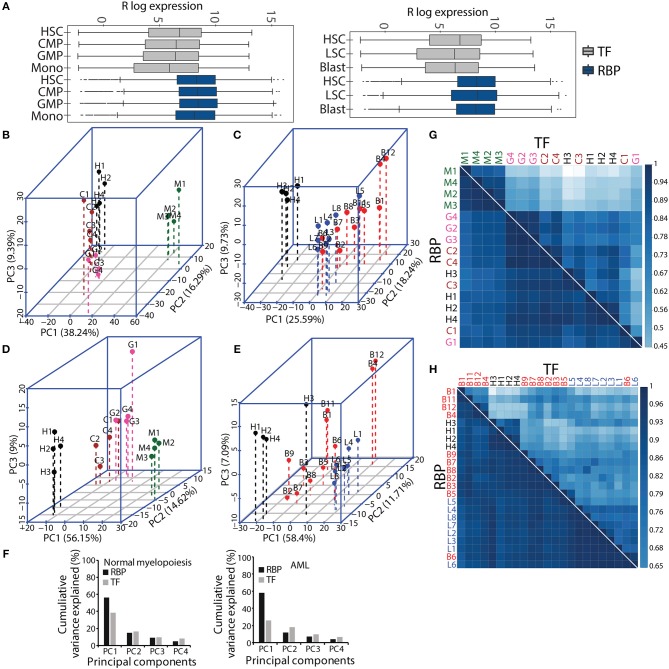
Comparative gene expression profiles of transcription factors (TFs) and RBPs in normal and malignant myelopoiesis. Box-whisker plot showing gene expression patterns of TFs and RBPs across normal myeloid differentiation (left panel) and leukemia (right panel). The *p*-values (Wilcoxon test) for the comparisons between TFs and RBPs for each cell type was <2.22e–^16^ **(A)**. Principal component analysis (PCA) showing the first three components for normal myeloid cell types (H-HSC, C-CMP, G-GMP, and M-monocytes) **(B,D)** and acute myeloid leukemia (L-LSC and B-Blast) **(C,E)** for TFs **(B,C)**, and RBPs **(D,E)**. Bar graphs showing percentage cumulative variance in the expression patterns of TFs and RBPs in normal myeloid differentiation and AML **(F)**. Spearman correlation matrix showing heat map to depict pairwise correlation coefficients for TFs and RBPs in normal and malignant myeloid cells, respectively **(G,H)**. The correlation plots and PCAs are based on gene expression cohort of 1,639 TFs, and 1,661 RBPs. The AML samples (LSCs and Blast) were compared to normal HSCs.

RBPs perform both diverse and redundant functions in regulating PTGR. However, some RBPs have been reported to be expressed in a tissue/cell type-specific manner (3). Hence, we were interested in correlating mRNA expression patterns/distribution of transcription factors (TFs) and RBP subclasses across the spectrum of normal and malignant myeloid cells. We compared the gene expression patterns of 1,639 human TFs, which are known to be expressed in lineage and cell type specific manner [(25); Supplementary Table 4] with that of RBPs in normal and leukemic cell types and observed that the overall median gene expression of RBPs was higher than that of TFs (Figure 2A). Comparison of the curated list of RBPs with that of TFs revealed a small overlap of 1.4% (Supplementary Figure 1A). Among the RBP subclasses, ribosomal RBPs (rRBPs) were expressed more than any other class of RBPs, possibly due to their role in translation related processes. We also observed that the transcript read counts of RBPs were low in monocytes (Supplementary Figure 1B). Interestingly, the overall read counts of all subclasses of RBPs in LSCs and blasts were similar to those in HSPCs (Supplementary Figure 1B).

Next, we performed PCA with the gene expression matrices for the TF and RBP genes to investigate the variance relationship in the four normal myeloid cell states (HSC, CMP, GMP and monocyte) and the two leukemic cell states (LSCs and blasts). The first three components of PCA scores incorporate maximum variance, which segregated different normal myeloid and leukemic cells based on TF and RBP gene expression. The expression patterns of TFs and RBPs yielded distinct clusters between HSCs, progenitors and differentiated monocytes in normal myeloid development and between HSCs and LSCs (Figures 2B–F). However, the leukemic blast cells did not form distinct clusters and were scattered in different components for both TF and RBP PCAs (Figures 2B–E). This result suggests that the transcriptional program goes “haywire” during LSC to blast transformation (26). We further observed that the RBP genes show distinct clusters between HSCs, CMP and GMP, with higher cumulative variance compared to TFs in component 1 (Figures 2D,F). Finally, we computed pairwise Spearman's rank correlation along with hierarchical clustering to determine the correlation between normal and AML cell types in terms of gene expression. The correlation plots between TFs clearly distinguished monocytes from HSPCs in normal myeloid cells, whereas HSCs and LSCs showed distinct correlation in the AML cohort. On the other hand, RBPs showed a higher correlation coefficient; however, compared to TFs, the distinction between the clusters was reduced in normal and AML myeloid cells (Figures 2G,H). Although RBPs perform ubiquitous functions, distinct cell type-specific expression clusters were still observed during myeloid differentiation. These results indicate that analysis of the expression profiles of RBP genes may provide important clues for understanding hematopoiesis and leukemia. In addition, this analysis suggested that RBPs are expressed in cell type specific manner both in normal myeloid development and in leukemia.

### RBP Gene Expression Can Distinguish RNA Dependent Processes During Normal Myeloid Differentiation

To understand changes in RBP gene expression during normal myelopoiesis, we have used two approaches, differential gene expression (DGE) analysis and gene-gene correlation analysis. DGE has been the method of choice to dissect transcriptome data; its major drawback is that only genes that are differentially expressed with an arbitrary fold change cut-off are considered relevant. Furthermore, DGE considers genes as individual independent functional units. In contrast, co-expression analysis is based on gene-gene correlation, and has been widely used to identify co-expressing/associating modules. Genes within a co-expression module, based on their co-relation indicates, that they might work in concert or possess opposing functions in similar biological processes (27, 28).

**Figure 3:**
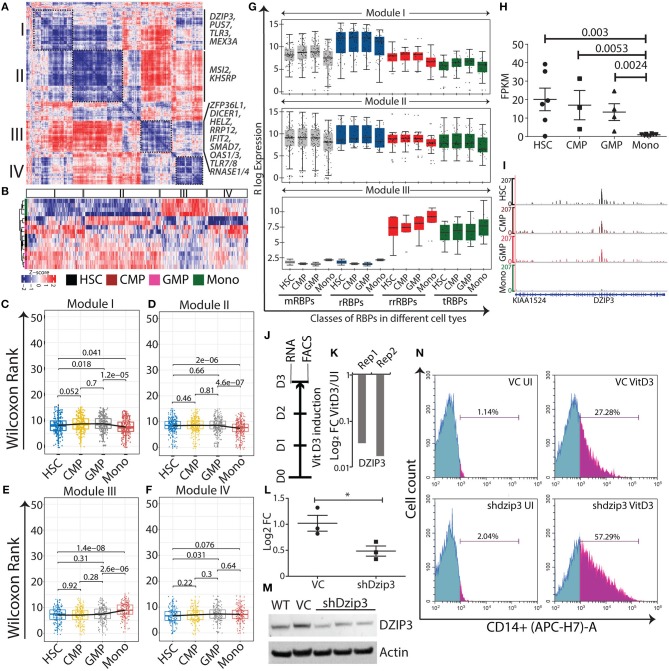
Normal myeloid cell-specific modules obtained from gene-gene correlation studies. Heat map showing Spearman correlation plot based on RBP gene expression values, and the black boxes show four modules that were considered for further studies **(A)**. Heat map representation of unsupervised clustering of RBP gene expression in different modules **(B)**. Box-whisker plots represent overall gene expression patterns of RBPs in HSC, CMP, GMP, and monocytes across four modules. Wilcoxon rank test shows *p*-values for each comparisons **(C–F)**. Box-whisker plots represent the gene expression pattern of RBP classes within modules I, II, and III; *p*-values for comparing the difference in each RBP class for different cell types is listed in Supplementary Table 5 **(G)**. Expression profile of DZIP3 was plotted from the bloodspot database using BLUEPRINT RNA-seq data **(H)**. Genome browser plots of DZIP3 from RNA-seq data analyzed in this paper **(I)**. Experimental workflow to identify the role of DZIP3 in U937 promyelocyte differentiation **(J)**. U937 cells were treated with 30 nM vitamin D3 for 72 h and DZIP3 transcript levels were assessed using qRT-PCR and plotted relative to actin levels in two biological replicates **(K)**. U937 cells were transduced with lentivirus containing shRNA against DZIP3, and 48 h post-puromycin selection, knockdown was confirmed both at the transcript level using qRT-PCR (relative to actin) **(L)** and at protein level using immunoblotting; actin was used as the loading control **(M)** from three independent experiments; error bars are mean ± s.e.m. *P < 0.05 per two-tailed Student's *t*-test. Representative histograms showing expression of monocyte-specific marker CD14 of three independent experiments. CD14 expression was assessed using flow cytometry, after vitamin D3 induction for 72 h in, vector control (VC) U937 (upper panel), and shdzip3 transduced U937 (lower panel) **(N)**.

Using the co-expression analysis, we have identified four distinct modules, I–IV, comprising 206, 273, 167 and 144 RBPs, respectively (Figures 3A–F and Supplementary Table 5). Expression of these RBPs in each module is shown in a heat map, which clearly demarcates the distinct cell type specific expression patterns of the RBP genes in different modules (Figure 3B). Furthermore, to understand the expression pattern of the modules within different cell states, we used non-parametric Wilcoxon rank test and plotted box-whisker graphs depicting the *p*-values for each comparison (Figures 3C–F). We observed that the expression of RBP genes was significantly higher in progenitors (CMP and GMP), HSC/progenitors and monocytes in modules I, II and III respectively, indicating their functional significance in a cell-type specific manner. Surprisingly, module IV consists of RBP genes with no significant difference in median expression. Pathway analysis using the reactome database (https://reactome.org/) revealed that RBPs in module IV were associated with RNA metabolism and translation-related processes (data not shown). Furthermore, we compared the expression of different classes of RBPs in the various modules. In module I, the majority of the classes, including mRBPs, rrRBPs and tRBPs, showed variation within the stem/progenitor compartment, with higher median expression in CMP and GMP than in HSCs and lower median expression in monocytes, emulating the overall module I expression pattern. Similarly, subclasses within module II are more consistent, with a comparable median in the stem progenitor compartment and low expression in monocytes. rrRBPs and tRBPs are the major contributors to module III and all the subclasses show higher expression in monocytes than in HSC, CMP and GMP (Figure 3G).

Simultaneously, we performed DGE analysis where we systematically compared HSCs with CMPs, GMPs and monocytes to identify differentially expressed RBPs (≥1.5 log_2_ fold change and false discovery rate (FDR) <0.05; ≥1.2 log_2_ fold change was considered for HSC and CMP comparison) (Supplementary Table 6). Unsupervised hierarchical clustering of DGE revealed four separate clusters (C1-C4) of RBPs with distinct expression patterns (Supplementary Figure 2A). The clusters have been named according to the type of myeloid cells with significantly higher RBP expression than other cell types in that particular cluster. C1, the hematopoietic stem/progenitor-specific cluster, consists of 109 RBPs and the C2, monocyte-specific cluster, contains 88 RBPs. The two smaller clusters, C3 and C4, with subsets of 12 RBPs showed progenitor-specific gene expression patterns, suggesting that these RBPs are potentially important for the progenitor phenotype (Supplementary Figure 2B). We compared the list of RBPs identified in a specific module with its corresponding cluster (by definition), to identify independent and overlapping information from co-expression analysis and DGE analysis (Supplementary Figures 3A,B). The majority of the genes identified in DGE clusters overlapped with the corresponding module. RT-qPCR validation of selected RBPs (from module I, II and III) in the HL60 cell line differentiation model showed that the expression patterns of several RBPs were similar to RNA-seq data (Supplementary Figure 4). Our systematic bioinformatics approach for understanding the potential functions of RBPs during normal myeloid differentiation led to the identification of several cell-type specific RBP. Next, we evaluated the functional relevance of these RBP genes within specific modules by curating information regarding these genes from existing literature.

### Functional Significance of the Distinct Expression of RBPs in Hematopoietic Stem Cells and Progenitors

Modules I (206 RBP genes) and II (273 RBP genes) consisted of RBPs specifically expressed in the HSC, CMP and GMP compartments, such as the previously reported hematopoietic stem cell regulators Musashi-2 (MSI2) and pseudouridylate synthase 7 (PUS7). MSI2 is highly expressed in HSCs and its expression decreases during differentiation into mature cells (29). In a similar manner, we observed a gradual reduction in the expression of MSI2 as HSCs differentiated into monocytes (log_2_ FC = −3.8), supporting their role in stem cell maintenance (Supplementary Figure 2C). Similar to MSI2, PUS7, a tRBP, is expressed more in HSPCs (log_2_ FC = −1.8), which decreases upon differentiation, strongly indicating its involvement in maintaining cell identity by regulating translation (30) (Supplementary Figure 5A). We also identified TLR3 and MEX3A as potential candidates for HSPCs maintenance and proliferation (Supplementary Figure 2C) which were downregulated with log_2_ FC of −7 and −7.8, respectively). In the developing brain, TLR3 is expressed in neuronal stem/progenitor cells and acts as a negative regulator of their proliferation by modulating sonic hedgehog signaling (31, 32). In contrast, MEX3A is required for maintaining intestinal stem cell homeostasis and it hinders differentiation by negatively regulating the expression of the TF, CDX2 (33, 34). Interestingly, CDX2 is ectopically expressed in patients with AML but not in HSPCs of normal individuals (35). These observations indicate that MEX3A and CDX2 are involved in normal and malignant myelopoiesis, which require further investigation.

### Functional Significance of the Distinct Expression of RBPs During Monocyte Differentiation

The monocyte-specific module III consists of 167 RBP genes, the expression of which increases concomitantly from HSPCs to monocytes (Figures 3E,G and Supplementary Figure 5). The regulatory RBPs enriched in this module has been previously reported to be involved in cellular differentiation during developmental processes. For example, SMAD7 (log_2_ FC = 2.6), a pleiotropic RBP that negatively regulates TGF-β signaling promotes myeloid commitment at the expense of lymphoid cells in cord blood multipotent progenitor cells that are enriched in this cluster [(36, 37); Supplementary Figure 5B]. ZFP36L1 (log_2_ FC = 3.4), a member of the ZFP36 zinc finger protein family member is a positive regulator of monocyte/macrophage differentiation (Supplementary Figure 5C). Mechanistically, it binds to AU-rich elements in the 3′ untranslated region (UTR) of the CDK6 mRNA and represses CDK6 expression, which is a negative regulator for monocytic differentiation (38). RNAse I and IV endoribonuclease and DICER1 were the other RBPs enriched in monocytes (Supplementary Figures 2C, 5D,E). HELZ and RRP12 are among the RBPs that potentially regulate monocytic differentiation were identified in this study (log_2_ FC of 0.6 and 2.01, respectively), but have not been reported previously for myeloid differentiation (Supplementary Figures 2C, 5F). We also detected RBP genes essential for immune-related functions of monocyte, such as IFIT2 (log_2_ FC = 1.9), which recognizes the 5′-triphosphate RNA and is required for generating an antiviral response [(39); Supplementary Figure 5G]. TLR7/TLR8 (log_2_ FC = 11 and 9.7, respectively comparison), which recognizes single-stranded viral RNAs and OAS1/OAS3 (log_2_ FC of 3.5 and 1.2, respectively), which recognizes double-stranded RNAs, the that are crucial for host immune responses [(40, 41); Supplementary Figures 5H,I]. Diverse classes of RBPs such as RNASE1 and RNASE4 (log_2_ FC = 4.8), which are associated with anti-viral and immune responses, are also upregulated in monocytes [(42); Supplementary Figures 5H,I] Taken together, these data suggest that cell stage-specific expression of key regulatory RBPs can potentially modulate myeloid differentiation program.

### DZIP3, a Potential Hematopoietic Stem Cell Factor

DZIP3 is a multifunctional RBP that contains RNA binding and E3 ubiquitin ligase RING domains. It represses differentiation-responsive genes in mouse embryonic stem cells by mediating changes in 3D chromatin conformation via H2AK119 ubiquitination (43). In our study, we observed DZIP3 to be a part the of HSPCs module II and is highly expressed in HSPCs and its expression decreased as HSCs differentiate into monocytes (2.3 log 2 fold upregulated). We selected DZIP3 for experimental validation as it has diverse functions as well as context-dependent roles as an epigenetic factor, transcriptional and post-transcriptional regulator. Importantly, the function of DZIP3 in hematopoiesis has not been investigated. We checked the expression of DZIP3 in “BloodSpot” database using BLUEPRINT RNA-seq data with default parameters, which showed a similar gene expression pattern [(44); Figure 3H]. Integrative genome plots of our analyzed RNA-seq further confirms this observation (Figure 3I). To validate the role of DZIP3 in cell proliferation and differentiation, we have used an *in vitro* U937 promyelocytic leukemic cell line-based differentiation model. The U937 cells were differentiated into monocytes by inducing the cells with vitamin D3 for 72 h (Figure 3J) and the level of DZIP3 transcripts in uninduced and differentiated U937 cells was determined. We observed that DZIP3 expression decreases upon induction, which corroborated the RNA-seq data of the primary cells where DZIP3 expression decreased in more differentiated cell progenies (Figure 3K). To understand the functional relevance of DZIP3 in HSPCs, we knocked down (KD) *DZIP3* using a lentivirus-based shRNA system in U937 cells and confirmed the reduction of *DZIP3* expression at both transcript and protein level (Figures 3L,M). The KD cells were subjected to vitamin D3-induced differentiation along with their respective controls. Cells expressing CD14, a monocytic differentiation marker, were scored using fluorescence-activated cell sorting (FACS). We observed significant enhancement of CD14 (2-fold) expression in *DZIP3*-depleted cells compared to the vector control cells (Figure 3N). The uninduced cells did not show any difference in CD14 marker expression. Our observations clearly indicate a role of DZIP3 in HSPCs maintenance, as loss of DZIP3 results in abrogation of the differentiation block upon induction.

### Expression of RBPs During Leukemic Transformation

**Figure 4:**
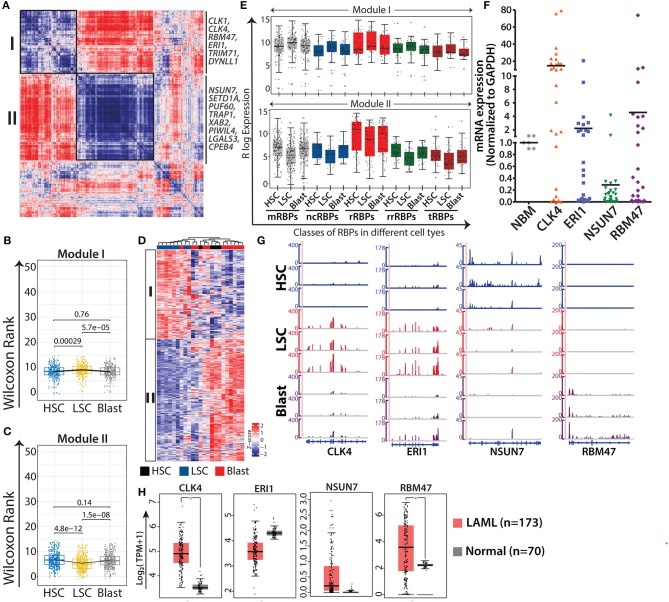
AML specific modules obtained from gene-gene correlation studies. Heat map showing Spearman correlation plot based on RBP gene expression values, and the black boxes show two major modules that were considered for further studies **(A)**. Box-whisker plots represent overall gene expression patterns of RBPs in HSC, LSC, and blasts across two modules Wilcoxon rank test shows *p*-values for each comparisons **(B,C)**. Unsupervised clustering of RBP gene expression in different modules **(D)**. Box-whisker plots showing expression of different classes of RBPs in module I (left) and module II (right); *p*-values for comparing the difference in each RBP class for different cell types is listed in Supplementary Table 7 **(E)**. qRT-PCR validations of the expression of selected RBPs, CLK4 (*p* = 0.0018), ERI1 (*p* = 0.1892), NSUN7 (*p* = 0.0001), and RBM47 (*p* = 0.1869) in samples from AML patients together with age-matched normal samples as controls **(F)**. Genome browser plots of *CLK4, ERI1, NSUN7*, and *RBM47* in individual samples of HSC, LSC, and blasts **(G)**. Box-whisker plot depicting the expression profiles of the same genes **(H)** in AML cohorts of TCGA and GTEx visualized in a GEPIA2 platform using default parameters (one-way ANOVA, *p* < 0.01). *p < 0.01.

LSCs sustain their leukemic nature by rewiring transcriptional and post-transcriptional programs and pose a major hurdle during therapeutic interventions. Considering the complexity of the RBP expression profile during normal myeloid differentiation, it is apparent that dysregulation of this axis will contribute to leukemic transformation and maintenance. Recent reports showed that dysregulation of the PTGR networks on RBP genes contributes to oncogenesis in leukemia (9, 11). To understand the RBP gene expression landscape during leukemic transformation, we performed both gene-gene correlation and DGE analysis in the context of normal myelopoiesis and compared HSCs with LSCs and leukemic blast cells. Gene-gene correlation studies revealed two major modules, I (303 genes) and II (394 genes) (Figure 4A and Supplementary Table 7). The RBP genes were extracted from the modules and their expression was plotted, which revealed that module I consisted of genes enriched in LSCs compared to HSCs and blasts, while module II consisted of genes that were downregulated in LSCs (Figures 4B,C). Heat map representation of the RBP genes from modules I and II showed high and low expression in LSCs respectively, and suggested that RBPs are involved in leukemogenesis (Figure 4D). We further analyzed the representation of different classes of RBPs in modules I and II to identify how the expression of different classes of RBP genes compare across HSCs, LSCs and blasts (Figure 4D). Interestingly, the median expression values of all RBP classes followed a trend similar to that shown in the box plots generated from average RBP expression values (Figures 4B,C,E).

Next, we validated the expression of selected RBPs, CLK4, RBM47 and ERI1 from module I, and NSUN7 from module II, in samples from patients with AML (*n* = 28) using qRT-PCR and compared the results with those obtained using age-matched normal bone marrow samples (Figure 4F). The results of qRT-PCR corroborated the expression pattern of these genes observed in the RNA-seq results (Figure 4G). Literature survey indicated that CLK4 and RBM47 are involved in RNA splicing, NSUN7 methylates enhancer RNAs, and ERI1 is a 3′-5′ exoribonuclease (45–47). CLK4 and NSUN7 showed significant upregulation and downregulation in samples of patients with AML, respectively, whereas ERI1 and RBM47 showed moderate changes (Figure 4E). The moderate differences in the expression levels of these genes between the results of qRT-PCR and RNA-seq can be attributed to the cellular heterogeneity within patient samples, which comprises blast cells, or to the purity of the FACS-sorted samples that were used for RNA-sequencing. Furthermore, we analyzed the expression of these genes in TCGA and GTEx cancer data sets from Gene Expression Profiling Interactive Analysis 2 (GEPIA2) (http://gepia2.cancer-pku.cn), along with the corresponding control RNA-seq data using default parameter (48). Results showed that all the genes followed similar expression pattern with significant upregulation of *CLK4, ERI1* and *RBM47* in AML cases compared to that in the control (Figure 4H).

DGE analysis of RBPs where HSCs were compared with LSCs and blasts revealed 332 differentially expressed RBPs (log_2_ fold change ≥1.5 and FDR ≤ 0.05) forming four distinct clusters based on expression (Supplementary Table 8). Heat map representation of unsupervised clustering of these genes revealed four different clusters of genes (Supplementary Figure 6A). Boxplots of Cluster C1 (30 RBPs) showed an increasing trend in the mean expression of RBPs from HSCs to LSCs and blasts. Cluster C2 (37 RBPs) was enriched in LSCs and Cluster C3 (48 RBPs) was enriched in RBPs expressed in HSCs. However, the largest Cluster C4 (217 RBPs) consisted of RBPs specifically downregulated in LSCs (Supplementary Figure 6B). This distinct pattern suggested extensive dysregulation of RBP gene expression during leukemic transformation. Overlap between RBP genes in the co-expression modules and the corresponding clusters are shown in Supplementary Figure 7.

### AML-Specific RBPs

We further investigated the RBP expression patterns of LSCs from module I, which contains a set of genes that are highly expressed in LSCs compared to in HSCs and blasts (Figures 4B–D). We detected expression of RBP genes such as *CLK1, CLK4, TRIM71, and DYNLL1* (1.6, 1.6, 1.75, and 2 log_2_ fold upregulated in LSCs, respectively), which have not been previously reported to associated with LSCs (Supplementary Figure 6C). The splicing kinases CLK1 and CLK4 were highly expressed in all the replicates of LSC, suggesting that this battery of splicing kinases might play essential roles in regulating the LSC-specific splice variants. Literature suggests that among these genes, *TRIM71*, encoding an E3 ubiquitin ligase, is highly expressed in undifferentiated ESCs and interacts with miR-302 and miR-290 to promote G1-S transition resulting in increased proliferation of ESCs (49). DYNLL1 is reported to be a critical regulator of B cell development and its overexpression is linked to Myc-driven B-cell lymphoma in a mouse model (50, 51). The biological functions of TMR71 and DYNLL1 in leukemic stem cell or AML blast cells are yet to be studied and hence these genes are potential candidates for further investigation.

The larger module II contained genes that are less enriched in LSCs, which was expected considering their significantly higher expression in HSCs and blasts. This module included RBP genes such as *SETD1A, PUF60, TRAP1* and *XAB2* (3.04, 2.9 and 3.7 log_2_ fold downregulated in LSCs, respectively) (Supplementary Figure 6C). SETD1A is a H3K4 methyl transferase harboring an RNA recognition motif and is essential for maintaining homeostasis of HSCs and for activation of DNA repair genes (52, 53). Other important candidates such as splicing factors PUF60 and XAB2 and TRAP1, a chaperone upregulated in various cancers and essential for maintaining mitochondrial homeostasis (54) were downregulated in LSCs.

RBP genes upregulated in blast cells compared to HSCs included *PIWIL4, LGALS3*, and *CPEB4* (3.5, 4.2 and 1.8 log_2_ fold, respectively) (Supplementary Figure 6C). PIWIL4 is involved in silencing of transposable elements via epigenetic modifications. Lack of PIWIL4 has been shown to induce differentiation in mouse erythroid leukemia (55). LGALS3 or galactin 3 is an endogenous lectin and regulates RNA splicing (56). A clinical study conducted on a cohort of 280 patients revealed negative correlation of galactin3 expression with poor outcome and overall survival (57). CPEB4 is essential for terminal erythroid differentiation, where it represses translation of several mRNAs including CDK6 and its own mRNA in a feedback loop (58). However, upregulation of *CPEB4* in blasts suggests that it might play antagonistic roles in leukemic conditions. Another putative regulatory RBP, HEXIM4, was also found in this cluster and has been reported to interact with the long non-coding RNA NEAT1 to form RNP complexes and regulate dsDNA-mediated innate immune responses and activation of type-I interferon (IFN) genes (59). Overall, RBPs enriched in this cluster may be important for understanding the mechanisms of differentiation block and uncontrolled proliferation, which are characteristic of blast cell physiology.

RBP genes that are enriched in module II may plausibly be involved in maintaining cellular functions in normal myelopoiesis and are dysregulated in leukemia. Examples include genes encoding ribosomal proteins RPL11, PSIP1 and membrane protein PTRF (1.54, 0.7, and 3.65 log_2_ fold downregulated in LSCs compared to in HSCs, respectively) (Supplementary Figure 6C). RPL11 is a ribosomal protein and PSIP1 forms a fusion transcript with NUP98 in myelodysplastic syndrome with *t*(9, 11, 60). Deletion of *PTRF* increases the number of LSKs and LT-HSCs in mice; however, these HSCs could not differentiate into mature cells (61). Overall, this suggests that RBP expression is significantly altered in normal hematopoiesis and leukemia, as indicated by the dysregulation in ribosomal and rRNA-related RBPs in the LSC compartment.

### Network Analysis Reveals Hubs of Ribosomal RBP Genes in LSCs

**Figure 5:**
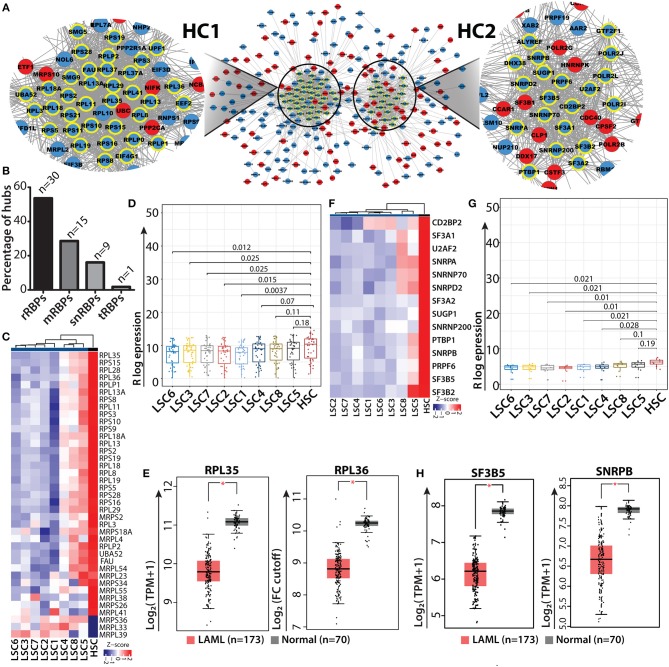
RBP interaction network and ribosomal RBP hubs in LSC. Shortest path gene interaction network of RBP and non-RBPs from module II; RBP hub genes are highlighted with bold yellow borders, and the color of the nodes depict the mode of expression of the gene in LSC compared to in HSC. Red denotes upregulated and blue indicates downregulated in LSC compared to HSC. Magnified view of a network depicting cluster of ribosomal proteins (hub-cluster 1 as HC1) and splicing related RBPs (hub-cluster 2 as HC2) **(A)**. Bar plot showing the percentage of hubs for different classes of RBPs; “n” represents number of RBPs **(B)** Heat map showing ribosomal RBP gene expression (*n* = 30) across individual LSCs compared to in HSCs (averaged expression values) **(C)**. Box-whisker plot (Wilcoxon rank test *p*-values) depicting overall downregulation of ribosomal proteins in LSCs **(D)**. Box-whisker plots showing significant downregulation of ribosomal hub RBPs RPL35 and RPL36 in AML from TCGA, GTEx data cohort visualized in GEPIA2 using default parameters; (one-way ANOVA, *p* < 0.01) **(E)**. Heat map and box-whisker plot (Wilcoxon rank test *p*-values) showing downregulation of splicing related RBPs (*n* = 14) in LSCs compared to HSCs **(F,G)**. Box-whisker plots showing significant downregulation of splicing related hub RBPs SF3B5 and SNRPB in AML from TCGA, GTEx data cohort visualized in GEPIA2 using default parameters; (one-way ANOVA, *p* < 0.01) **(H)**. *p < 0.01.

Gene interaction networks provide systematic understanding regarding the mechanisms via which genes communicate with each other and relay functional information within the network. The shortest path approach is used in these gene networks to prioritize vertices i.e., hubs which might be important in the biological system to predict functional modules and pathways and to predict functions of the vertices, i.e., genes within the network (62–66). To understand how RBP genes communicate with one another, we performed network analyses of gene interactions and traced the shortest paths of interactions between two RBP genes. The resulting networks consisted of the shortest possible routes via which RBP genes can communicate with each other via several intermediate genes (or nodes), many of which were non-RBP genes. We identified the “hub” genes with significant central importance in the network, which were highly connected to many other genes within the interactome. The higher the degree of connectivity of a hub gene, the more is its influence within the network and on the various associated biological processes. Many of the RBP genes were identified as hub genes in the networks and were shown to interact with multiple non-RBP genes (Supplementary Table 9). Several of these hub RBP genes have also been reported as important functional regulators of normal and malignant hematopoiesis (9). We highlighted the direct interacting neighbors (also called the 1-hop neighbor) of hub RBPs in the networks. Majority of the hub RBPs consisted of ribosomal RBPs (rRBPs) (30 out of 55 total hub RBPs), which included both cytoplasmic large and small ribosomal subunit proteins (Figures 5A–C). Heat map and box-whisker plots of all the ribosomal RBPs present in module II depict uniform downregulation of these RBPs in all individual LSC patient samples compared to in HSCs (Figures 5D,E). Hence, we mined the literature to understand how rRBPs potentially affect normal myelopoiesis for leukemic transformation. Cai et al. (67) have shown that RUNX1 deficiency is associated with proliferative advantage for HSPCs, transforming them into pre-leukemic stem cells. Further analysis revealed that RUNX1 directly binds to the promoter of ribosomal genes and activates them. Thus, in the absence of RUNX1, ribosome biogenesis is disrupted, which decreases translational rate along with acquisition of resistance to genotoxic and endoplasmic reticulum stress (67). This study provides direct evidence that low expression of ribosomal genes observed in data might correspond to reduced ribosomal biogenesis and protein synthesis. This could be a general mechanism for acquiring stress resistance by LSCs of different origins or genetic backgrounds. Next, we asked how the expression profiles of representative hub rRBPs are downregulated in LSCs of the larger AML cohort. We observed significant downregulation of *RPL35* and *RPL36*, structural proteins of the large ribosomal subunit, in TCGA and GTEx data compared to the controls in the GEPIA2 platform using default parameters [(48); Figure 5E]. We also observed a similar trend of downregulation of ribosomal and splicing related hub *RBPs* namely, *RPL7A, RPL11, RPL18* and *RPS11* compared to that in the control (Supplementary Figure 8A). HSCs are characterized by lower protein synthesis rate than committed progenitors and differentiated cells (68). LSCs might utilize similar mechanisms for their survival and contribute to disease relapse.

In addition to rRBPs, a significant number of RBP genes related to the splicing machinery (14 out of 55 total hub RBPs) were identified in network analysis. The heat map and box whisker plot show low expression of these genes across all the LSC samples with respect to HSCs (Figures 5F,G). We wanted to see if these splicing factors which were identified as hubs show any significant difference between HSC and LSC comparison. Therefore, we categorized hub genes from HC2 into low and high expression groups, which were determined from the lower and upper interquartile range and non-parametric test for both the groups. We found 8 RBP genes in the low expression group show significant differences in the mRNA levels with *p* < 0.05. These RBPs were associated with either U1 (SNRPA and SNRNP70) or U2 (SF3A2, SF3B5 and SNRPB) snRNP complex, which is essential for all U1/U2-dependent splice site recognition of the spliceosomal complex (69). We interrogated the AML data cohort of TCGA and GTEx in GEPIA2 platform and observed significant downregulation of SF3B5 and SNRPB in AML patients compared to control (one-way ANOVA, *p* < 0.01) (Figure 5H). We also observed a decreasing trend in the median expression of SF3A2, CD2BP2, SNRPA and U2AF2 (Supplementary Figure 8B). Similar analysis was carried out with RBPs present in module I, where most of the RBPs have high mRNA levels in LSC. The hub RBPs present from module I were of different classes as shown in Supplementary Figure 9.

## Discussion

In this study, we have elucidated a comprehensive landscape of RBP expression across myeloid developmental stages and discussed their dysregulation during leukemic transformation. Similar to transcription factors, RBP expression profiles can segregate normal myeloid cell types (HSCs, progenitor cells and monocytes) and leukemic cells (HSCs, LSCs and blasts), indicating that their functions are myeloid development stage-specific. We performed DGE and gene-gene correlation analysis to uncover the distinct RBP gene expression profiles in normal myelopoiesis and AML. The unbiased gene-gene correlation analysis revealed cell/stage-specific modules containing different classes of RBPs and provided information regarding both their abundance and expression patterns in different myeloid cells. This analysis revealed several RBPs, the functions of which have been previously implicated in normal myelopoiesis and AML biology. At the same time, we also detected RBPs that have not been implicated in normal or malignant hematopoiesis and may therefore act as novel candidates for further investigations regarding their function in myelopoiesis. Network analysis identified several rRBP genes as hubs within the gene interaction network, the expression of which is lower in LSCs than in HSCs. Finally, we strengthened our observations by investigating the expression profiles of RBP genes in other data cohorts and experimental verification of the expression of selected RBP genes using leukemic cell line differentiation models. We showed that DZIP3 is essential for HSCs, as loss of its expression enhanced differentiation of leukemic cells to monocytes upon induction. In addition, we validated the gene expression of *CLK4, NSUN7, ERI1* and *RBM47* in samples from AML patients. Taken together, our study highlights the importance of RBPs in normal and malignant myelopoiesis; furthermore, this study will act as a resource for the scientific community for further investigations on the mechanism of action of RBPs.

RBPs are the guardians of the post-transcriptional gene regulatory machinery and are involved in the regulation of all biological processes, alterations of which are associated with various malignancies. McKee et al. used *in situ* hybridization to show that 323 of 380 RBPs were expressed in a region-specific manner in the post-mitotic and proliferative zones of the brain (70). Recent studies have shown that RBPs are frequently mutated and are mostly downregulated across a diverse array of cancers (71). Several reports show that RBPs are involved in fine-tuning of self-renewal, lineage choices and differentiation of HSCs. Therefore, dysregulation of this axis manifests as blood-related disorders such as myelodysplasia and leukemia. We evaluated our analysis by comparing the RBPs in normal and AML modules with that in the COSMIC and LGL databases (Supplementary Figure 10 and Supplementary Table 3). Interestingly, most of the RBPs described in the Results are unique to normal and AML modules and are not listed in the databases. RBP genes such as *DICER1, BRCA1* and *PSIP1* were associated with all the intersections. Thus, the overlapping intersections revealed several RBPs that have previously not been implicated in AML pathogenesis and warrants further investigations.

PTGR by RBPs adds another level of regulatory complexity that dictates the fate of transcripts and translation efficiency. Gerstberger et al. (3) showed that 6% RBPs of the mRBP and ncRBP category show tissue specificity. They also identified several classes of RBPs to be co-expressed during brain and ovary development, which suggests that RBPs can be cell/tissue-specific (3). In this study, PCA analysis provided the first indication regarding the specificity of RBPs, where distinct clusters of HSCs, progenitors and monocytes were observed in normal myelopoiesis and of HSCs, LSCs and blasts in AML samples. Furthermore, the box plots for normal myelopoiesis showed modules I, II and III with high expression of RBPs in progenitors, HSPCs and monocytes, respectively, whereas the two modules identified in AML samples had either higher or lower expression of RBPs in LSC than in HSCs and blasts. Differential gene expression analysis also identified distinct expression profiles of RBPs with several distinct clusters. These findings support our hypothesis that RBPs perform distinct functions in myeloid development and AML pathogenesis, which warrants further investigations.

Bioinformatics analysis for normal myelopoiesis revealed that several RBP genes were involved in HSPCs maintenance or monocyte differentiation. In agreement with the results of previous studies, we observed that *MSI2* expression was high in HSCs. MSI2 binds to the 3′ UTR of mRNA and acts as a translational repressor that is highly expressed in hematopoietic stem cells; its expression decreases with differentiation of HSCs into mature cells (29). In mice, lack of MSI2 significantly reduces the number of bone marrow HSCs and combined with severe defects in engraftment upon serial transplantation, this suggests that MSI2 is essential for self-renewal and maintenance of long term (LT)-HSCs in mice (72). Higher expression of MSI2 is linked with aggressive cancer and poor prognosis of AML patients with normal karyotype (26). Another RBP, PUS7 was enriched in HSPCs suggesting it to be an important stem/progenitor factor. PUS7 mediates the pseudouridylation of small RNA derived from tRNAs and releases 5′ terminal oligoguanine (TOG), which is a potent translational repressor. PUS7 knockout in HSPCs (CD34^+^) impairs translation and reduced differentiation, which was rescued by addition of TOG to the system. Furthermore, reduced expression of PUS7 has been implicated in hematological malignancies and clonal disorders. DZIP3, a multifaceted RBP was also enriched in HSCs, similar to MSI2 and PUS7. We show that *DZIP3* knockdown enhanced monocytic differentiation, which was in agreement with the results of a published report showing DZIP3 to be critical for the repression of differentiation-related genes (43). In a separate study, the RBD of DZIP3 has been reported to interact with HOTAIR lncRNA to regulate senescence in human fibroblasts by ubiquitinating and degrading Ataxin-1. In addition to domain-specific functions, DZIP3 interacts with coactivator-associated arginine methyltransferase 1 (CARM1) and acts as transcriptional co-activator of ERα-responsive genes (73). Till date the functions of DZIP3 in myeloid differentiation have not been studied and hence it warrants further investigation. The monocyte-specific RBP, module III, also contains several RBPs one of which is DICER1, an essential component of miRNA biogenesis. Our observation was supported by the results of a previous study showing that DICER1 ablation in the myeloid progenitor compartment (GMP) leads to myeloid dysplasia and impaired monocytic/macrophage differentiation by regulating differentiation-associated miRNA (74, 75). These support our observations but also highlight the contribution of RBPs in myelopoiesis by shaping the transcriptome of cells via stabilization or degradation of specific transcripts concurrent with stage-specific cues.

Owing to perturbations in their transcriptional and post-transcriptional program, LSCs constitute a major challenge to the success of therapeutic interventions and is the cause of chemoresistance and disease relapses. LSC-specific gene expression correlates with poor prognosis in AML (9). Several snRBPs, mRBPs (splicing factors) and post-transcriptional RNA modifiers have been strongly implicated in leukemic transformation (76, 77). Here, we have identified 303 and 394 RBPs with LSC signature (high expression) and anti-signatures (low expression) respectively in AML. Interestingly, these signatures were primarily present within mRBPs, ncRBPs, rRBPs, rrRBPs and tRBPs, suggesting that these RBPs play major roles in leukemic transformation. However, the mechanism via which these RBPs regulates the onset and maintenance of AML is not known, which is also the limitation of this study. Enhanced expression of splicing kinases CLK1, CLK4 and RBM47 in LSCs indicates that they can be potential therapeutic targets for drug-resistant and recurring cases of AML.

Network analysis uncovers unknown regulatory circuits within a gene interactome. In this study, we identified a densely connected module of rRBPs and U1/U2 snRNP related RBPs, several of which act as hubs within the network. Compared to HSCs, we observed downregulation of these rRBPs and splicing related RBPs in LSCs, which is indicative of a cancer stem cell-specific, post-transcriptional gene regulatory network coupled to low protein synthesis, low profile translatome and altered splicing events. Reduction in protein synthesis can contribute to cancer stem cells in different ways; for example, it can affect protein turnover and lower the chances of producing tumor antigens, thereby evading the immune system (67). Low ribosomal activity has been shown to promote translation of a pool of oncogenic mRNA transcripts required for sustaining the oncogenic program (78). Thus, our study highlights that the RBP-mediated circuit of gene interactions is a critical functional axis in LSCs, dysregulation of which disrupts various oncogenic programs essential for LSC function. Overall, we have cataloged several RBPs essential for normal and malignant myelopoiesis, with focus on LSCs. This study lays a framework for studying the mechanisms via which RBPs may regulate normal or malignant hematopoiesis. Although our study has given vital insight into the potential role of RBPs in hematopoiesis, the study also has certain limitations. First, co-expression analysis for determining gene function is associated with computational and bioinformatics limitations. For example, co-expressed genes may not have related functions, and conversely, genes with related function may not be co-expressed because of post-transcriptional regulation. In addition, the threshold of expression similarity used may lead to over or underrepresentation of co-expressed genes. Second, the type, size and number of datasets used for the comparative analysis may also affect the output. Finally, owing to the large number of clustering algorithms that are currently in use, there is no single best method and parameter choice may affect the types of co-expression clusters obtained (79). It is essential to incorporate various layers of “omics” analyses, such as comparison of cross-linking immunoprecipitation (CLIP)-seq data together with transcriptome data to completely deconstruct the intricate architecture of transcriptional and post-transcriptional gene regulation by RBPs and enhance our understanding of normal myeloid differentiation and leukemia.

## Data Availability

The datasets analyzed for this study were downloaded from the Gene Expression Omnibus (GEO) (GSE74246) (https://www.ncbi.nlm.nih.gov/geo/query/acc.cgi?acc=GSE74246) and was originally generated by Corces et al. (13).

## Ethics Statement

Human bone marrow aspirates were obtained from Park Clinic, Kolkata, India, from untreated, patients newly diagnosed with AML. Informed consent was obtained for all patients. The study protocol was approved by Institutional Human Ethics Committee and was performed per guidelines of the Council of Scientific and Industrial Research–Indian Institute of Chemical Biology (CSIR-IICB) Institutional Review Board as reported previously in Boila et al. (19), Chatterjee et al. (20) and Biswas et al. (21).

## Author Contributions

PP has conceptualized the project and secured funding. KM and SS have performed majority of the bioinformatics analysis, including data processing for RNA-sequencing. SS performed majority of the wetlab work. MB and AS have validated AML patient samples using qPCR. SChak performed network analysis. JB and SM performed qPCR validation in cell line model. SChau and SK have helped in optimizing and western blotting of DZIP3. PP and SS prepared the figures and wrote the manuscript. PP, SS, and SChak edited the manuscript.

### Conflict of Interest Statement

The authors declare that the research was conducted in the absence of any commercial or financial relationships that could be construed as a potential conflict of interest.
