# Supplementary material for: Transcriptomic Analysis Identifies RNA Binding Proteins as Putative Regulators of Myelopoiesis and Leukemia
Source: Front Oncol. 2019 Aug 6;9:692. doi: 10.3389/fonc.2019.00692 (PMC6691814; doi:10.3389/fonc.2019.00692)
Supplement: Supplementary file 11 [file Data_Sheet_1.docx]

**Supplementary Data**

1. **Correlating the expression of curated transcription factors (TFs) and RNA binding proteins (RBPs).**The curated TF and RBP genes were compared to identify the RBPs that behave as TFs. We observed 1.4% (46 out of 1661 RBPs) overlap between genes encoding TFs and RBPs, suggesting that these RBPs can also act as TFs. We next examined the expression of TFs and RBP subclasses across the normal myeloid cells and leukemic cells. The median expression of all the RBP sub-classes was higher than that of TFs in all cell types, highlighting their importance in regulating PTGRs.


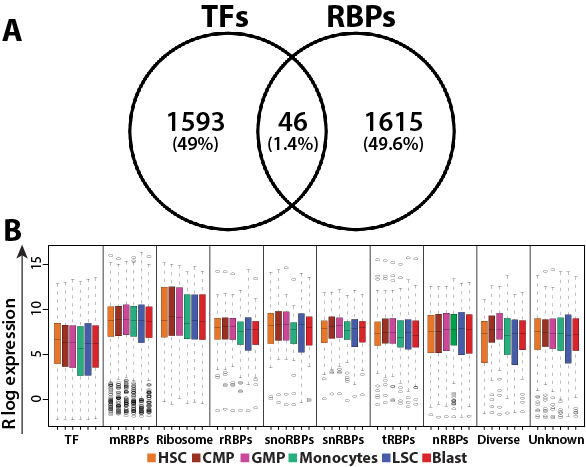


**Supplementary Figure 1: Correlating the expression of TFs and RBPs.** Venn diagram showing overlap of genes between curated list of 1,661 RBPs that were identified in the analyzed RNA-seq data and 1,639 TFs expressed in the hematopoietic system (A). Box-whisker plot showing TFs and the expression of different classes of RBPs across normal and myeloid cell types (B).

**2. Differential gene expression analysis of RBPs in normal myeloid development.** We compared normal myeloid cells, namely, CMP, GMP, and monocytes individually with HSCs to identify the differentially expressed genes in each comparison. We used HSCs as our baseline reference for comparison as other blood cells are derived from HSCs. Unsupervised hierarchical clustering of 221 differentially expressed genes across all comparisons revealed clusters (C1-C4) with similar RBP expression pattern.


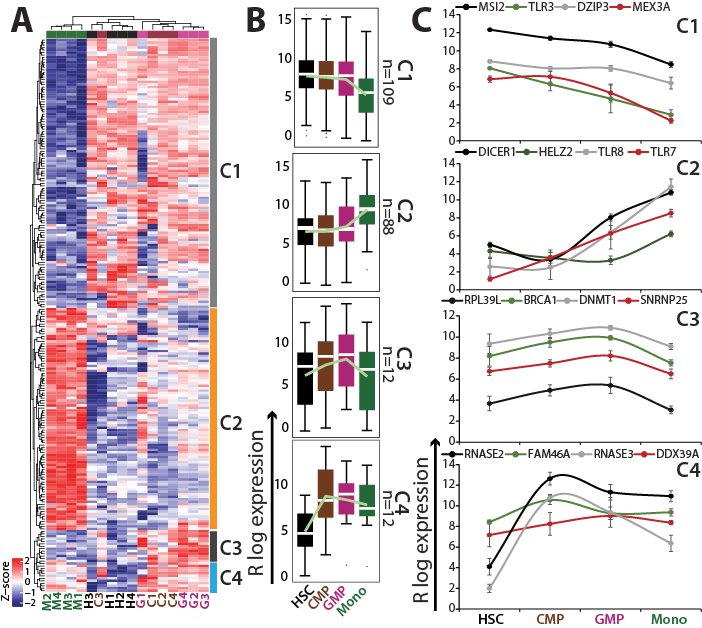


**Supplementary Figure 2: Differential gene expression analysis of RBPs in normal myeloid differentiation.** Heat map showing ribosomal RBP expression across individual HSCs, CMPs, GMPs, and monocytes segregated in clusters C1, C2, C3, and C4 (A). Box-whisker plot depicts expression profiles of each cluster with averaged replicates; connecting lines indicate moving averages and dashes indicate median values (B). Line graph showing the expression profiles of selected RBPS from each cluster; error bars show mean deviation (C).

**3. Comaprision of DGE and co-expression analysis in normal hematopoiesis.** We have undertaken two independent approaches to dissect the transcriptomic data and identify the RBP gene expression landscape across myeloid differentiation and leukemia. Co-expression analysis reveals modules of genes clustered together based on similar or co-ordinated expression. DGE analysis considers only genes that have passed an arbitarty criteria of fold change (which is calculated for each gene individually across samples). However, genes do not act independent of each other in a biological system. Genes that are related to the same biological process such as signalling, or are part of the same marcomolecular complex, tend to co-express. Therefore, co-expression analysis is important while considering RBPs, as most of them interact with RNA and other proteins to form functional RNP complexes such as ribosome, spliceosome, and so on (Yang et al., 2013; van Dam et al., 2018).

Here, we have performed comparative analysis of the RBP gene information obtained using DGE and co-expression analysis to asertain that significant information is not lost. Hence, we compared modules and clusters based on gene expression patterns. The RBP genes in module I and II were compared with those in Cluster I because they were enriched in genes expressed in HSC, CMP, and GMP. RBP genes in module II were compared with those in Cluster II, both of which contained monocyte-specific genes. In both cases, we observed that the majority of the genes identified in DGE clusters I and II were a subset of co-expression modules I, II, and III.


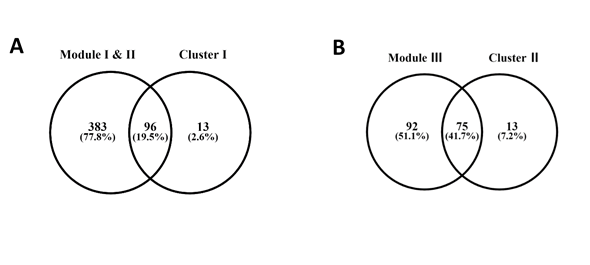


**Supplementary Figure 3:** **Overlapping and unique RBPs identified using DGE and co-expression analysis for normal myeloid differentiation.** Venn diagram of RBP genes in modules I and II, and cluster III (A), and in module III and cluster II (B), showing the number of overlapping and independent RBP genes identified using co-expression and DGE analysis.

**4. Quantitative PCR validation of selected RBP genes from analysis in the HL-60 differentiation model**. Briefly, HL60 is a leukemic cell line blocked in the promyelocytic stage. It can be efficiently differentiated into monocytes by treating with 50 nM 1α,25-dihydroxyvitamin D3 for 72 h. Differentiation was confirmed using flow cytometric assessment of the monocytic maker CD14 (data not shown) and also by detecting the mRNA level using qPCR. We used this model to examine the expression of selected RBPs from our analysis. We observed significant upregulation (> 1.5 log_2_ FC compared to wild type HL60 cells) of genes from module III, which comprised monocyte-specific RBP genes (Figure 4). However, among the genes selected from modules I and II (RBP genes enriched in the stem/progenitor compartment), we were able to validate the expression of only TERT, which was significantly downregulated (< 1.5 log_2_ FC compared to wild type HL60 cells) (Figure 4). Other genes did not show any significant difference, which can be partially explained by the fact that HL60 is a leukemic cell line, in vitro differentiation of which might differ from the normal course of HSC/P differentiation.

**Supplementary Figure 4: qPCR validation of selected RBPs and the monocytic marker CD14 in the HL60 differentiation model. Fold change of s**elected RBPs obtained from RNA-seq data (A) and qPCR of those genes in HL60 in vitro differentiation model. Log_2_ fold change were plotted with respect wild type HL60 cells from three independent experiments; error bars indicate S.E.M. (B)

**5. Box-plot of module-specific genes from RNA-seq data.**  The expression values of RBP genes discussed in the main text have been plotted in HSC, CMP, GMP, and monocytes.


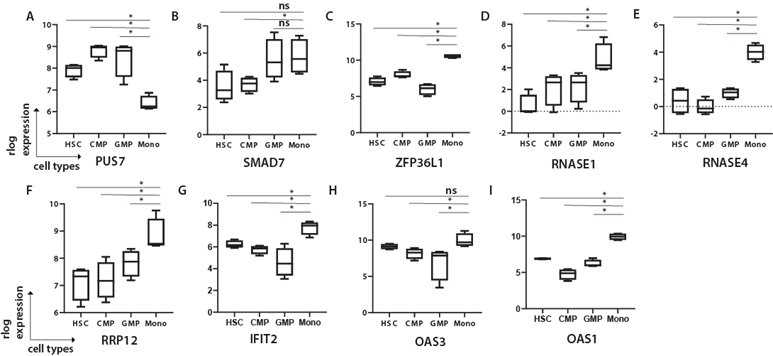


**Supplementary Figure 5: Expression of specific RBP genes from RNA-sequencing data.** rlog transformed expression values of *PUS7* (A), *SMAD7* (B), *ZFP36L1* (C), *RNASE1* (D), *RNASE4* (E), *RRp12* (F), *IFIT2* (G), *OAS3* (H), and *OAS1 (I)* from RNA-sequencing data plotted in HSC, CMP, GMP, and monocytes. Mann Whitney one tailed test; * *p* value < 0.03.

**6. Differential gene expression analysis of RBPs in normal myeloid development.** We compared LSCs and blasts with HSCs to identify the differentially expressed genes in each comparison. Unsupervised hierarchical clustering of 332 differentially expressed genes across all comparisons revealed clusters (C1-C4) with similar RBP gene expression pattern.


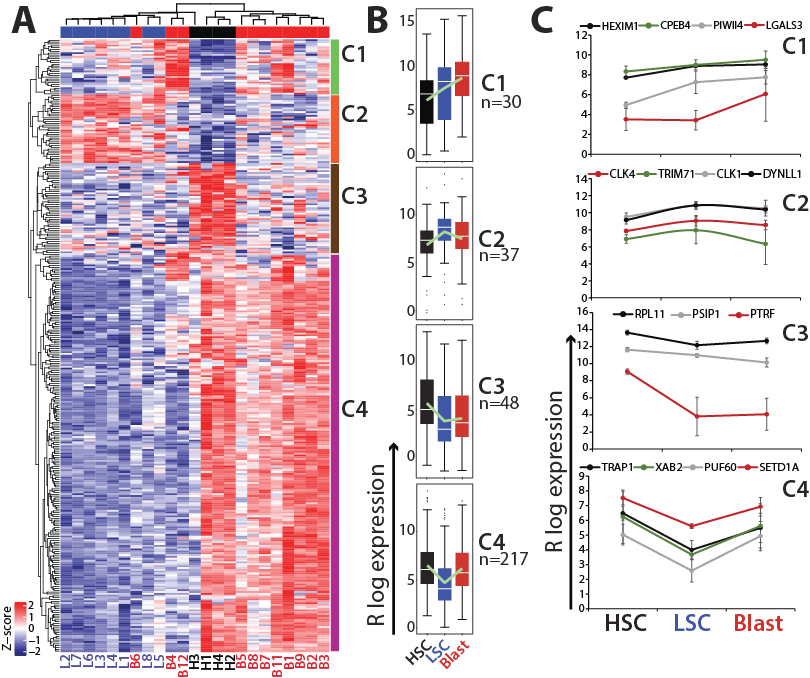


**Supplementary Figure 6: Differential gene expression analysis of RBPs in AML.** Heat map showing ribosomal RBP gene expression across individual HSCs, LSCs, and blasts segregated in clusters C1, C2, C3, and C4 (A). Box-whisker plot depicting the expression profiles of each cluster with averaged replicates; connecting lines indicate the moving averages and dashes indicate the median values (B). Line graph showing expression profiles of selected RBPS from each cluster; error bars show mean deviation (C).

**7. Comaprision of co-expression and DGE analysis in AML.** We performed a comparative analysis of the RBP gene information obtained from DGE and co-expression analysis in HSCs, LSCs, and blasts. The RBP genes in module I were compared with those in Cluster I as they were enriched in genes expressed in LSCs. RBP genes in module II were compared with those in Cluster IV as both contained monocyte-specific genes. In both cases, we observed that the majority, i.e., 91% and 94% of the genes identified in DGE clusters I and II, respectively, were a subset of co-expression modules I, II, and III. In case of leukemia, DGE analysis revealed two more clusters, I and III, which were enriched in blasts and normal HSCs, respectively. Significant modules mirroring such patterns were not identified using co-expression analysis. Hence, we concluded that the combination of both the approaches is essential for dissecting transcriptomic data.


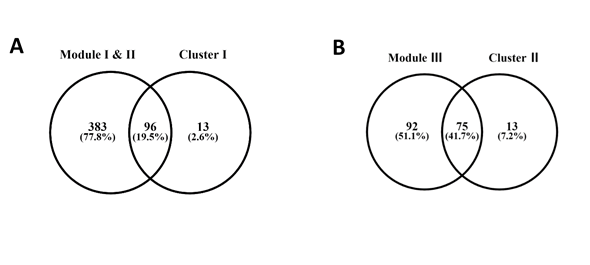


**Supplementary Figure 7:** **Overlapping and unique RBPs identified from DGE and co-expression analysis for AML datasets.** Venn diagram of RBP genes in module I and cluster III (A), and module II and cluster IV, showing the number of overlapping and independent RBP genes identified using co-expression and DGE analysis.

**8. RBP hubs depict a decreasing trend in expression in AML patients.** The expression of ribosomal RBP genes from HC1 and splicing-related RBPs from HC2 were interrogated in TCGA and GTEx AML cohort (N = 173) and compared to the control (N = 70) in GEPIA2.


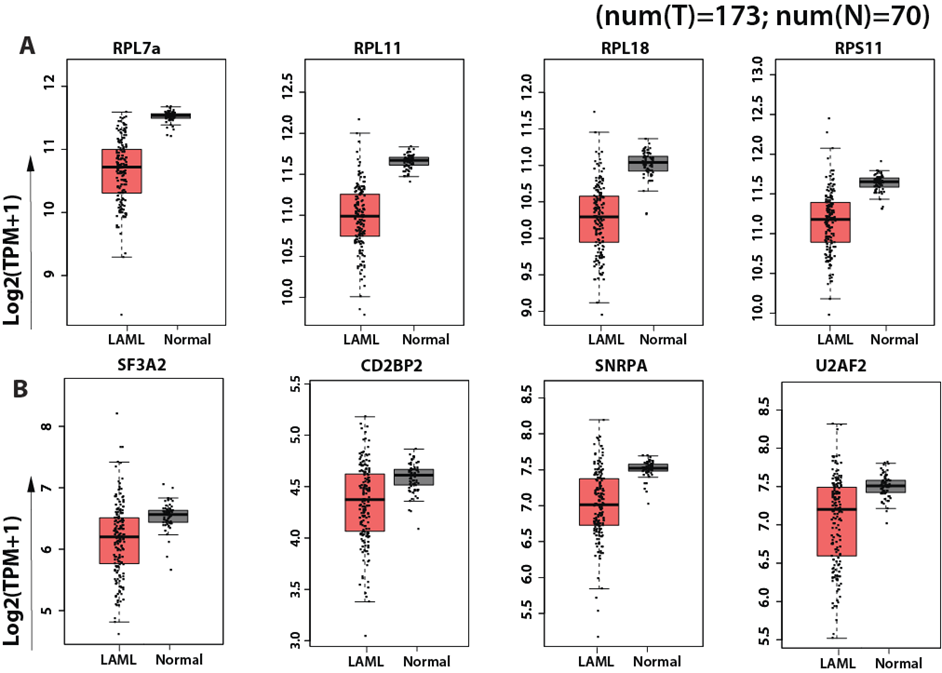


**Supplementary Figure 8: Extraction of RBP hub gene expression patterns from GEPIA2.** Box-whisker plots showing the expression patterns of selected ribosomal RBPs (RPL7A, RPL11, RPL13, RPL18, and RPS11) and splicing-related RBPs (SF3A, CDBP2, SNRPA, U2AF2) identified as hubs from network analysis, in AML TCGA, and GTEx data cohort visualized in GEPIA2 using default parameters (A, B).

**9. Network analysis of RBPs from AML module I.** We followed an approach similar to that mentioned in section 3.9 of the main text, to understand the RBP interactions in AML RBPs of module I. We observed that several transcription initiation factors acted as hubs.


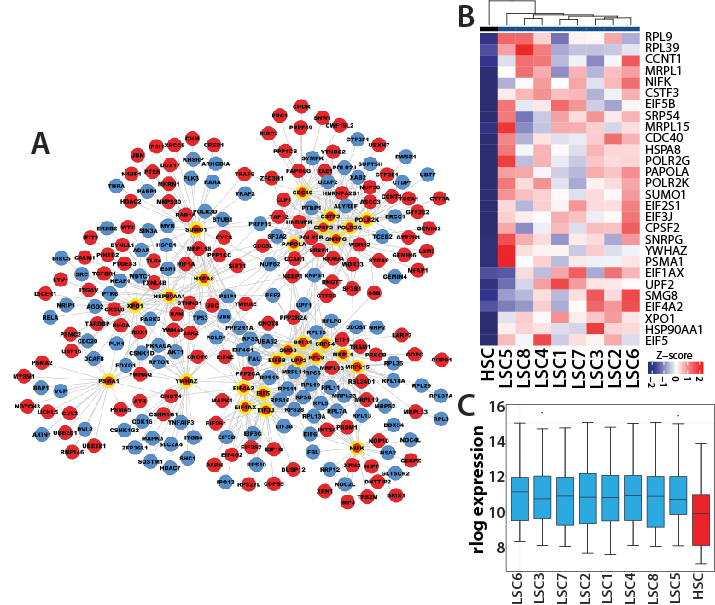


**Supplementary Figure 9: Shortest path network analysis for RBPs from AML module I.** Shortest path gene interaction network of RBP and non-RBPs from AML module I; RBP hub genes are highlighted with bold yellow borders, and the color of the nodes depict the mode of expression of the gene in LSCs compared to in HSCs. Red denotes upregulated and blue indicates downregulated in LSCs compared to in HSCs. Heat map showing hub RBP gene expression across individual LSCs compared to in HSCs (averaged expression values) (B). Box-whisker plot depicting overall downregulation of hub genes in LSCs (C).

**10. Comparative analysis of RBPs from normal myeloid and AML modules with COSMIC and leukemic gene literature database (LGLD).** To determine the number of RBPs that have been implicated in leukemogenesis till date and the unique RBPs from our study, we have compared RBP genes from different modules with that in cancer databases. We have selected Catalogue of Somatic Mutations in Cancer (COSMIC) (<https://cancer.sanger.ac.uk/cosmic>) and the LGLD database (<http://soft.bioinfo-minzhao.org/lgl/index.html>) (Liu et al., 2018) because the former provides the list of RBPs involved in different cancers, while the later provides information on RBPs implicated in AML. We identified 82 and 134 RBPs listed in COSMIC and LGLD out of 1,734 RBPs from the curated list and compared their association with RBPs of normal myeloid modules (I-IV) and AML modules (I-II). The UpSet plot below shows intersections and the number of RBPs overlapping for each comparision (Conway et al., 2017). Lack of intersection indicates unique subset of RBPs in each data set. This analysis identifies both overlapping and unique RBPs that are potentially involved in normal myeloid differentiation and AML. A complete list of RBPs present in all the intersections is shown in Supplementary Table 3. A detailed investigation is essential for dissecting the functions of RBPs in leukemogenesis.


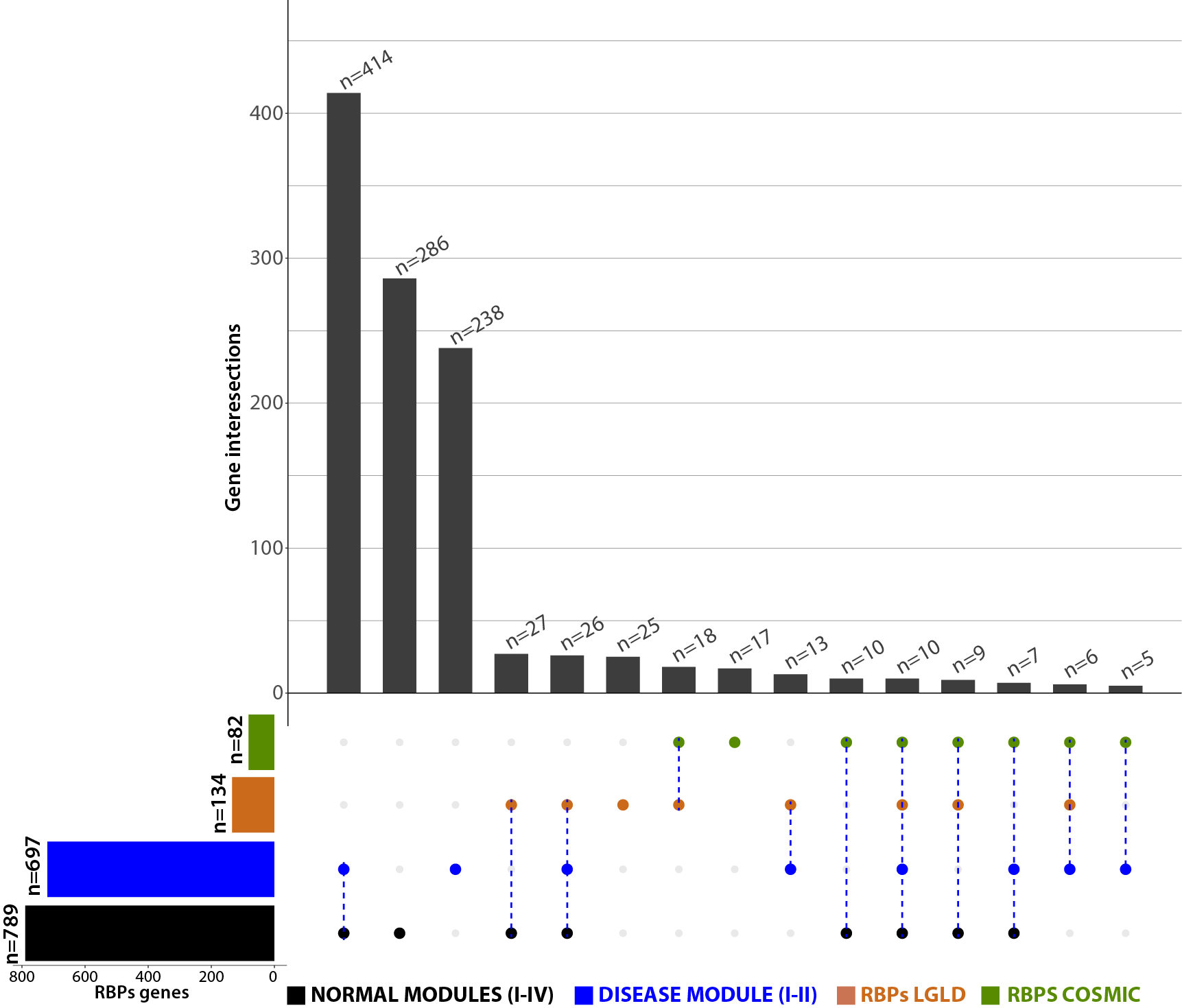


**Supplementary Figure 10: UpSet plot showing the intersections between normal, AML, COSMIC, and LGL databases. The** total number of RBPs from gene-gene correlation analysis for both normal and AML were compared with the COSMIC and LGL databases. The upset plot shows various intersections (dotted blue lines) indicating the number of RBPs identified for various comparisons. The unique RBPs present in four datasets are shown in solid circles.

**References:**

Conway, J.R., Lex, A., and Gehlenborg, N. (2017). UpSetR: an R package for the visualization of intersecting sets and their properties. *Bioinformatics* 33(18)**,** 2938-2940. doi: 10.1093/bioinformatics/btx364.

Liu, Y., Luo, M., Jin, Z., Zhao, M., and Qu, H. (2018). dbLGL: an online leukemia gene and literature database for the retrospective comparison of adult and childhood leukemia genetics with literature evidence. *Database (Oxford)* 2018. doi: 10.1093/database/bay062.

van Dam, S., Vosa, U., van der Graaf, A., Franke, L., and de Magalhaes, J.P. (2018). Gene co-expression analysis for functional classification and gene-disease predictions. *Brief Bioinform* 19(4)**,** 575-592. doi: 10.1093/bib/bbw139.

Yang, E.W., Girke, T., and Jiang, T. (2013). Differential gene expression analysis using coexpression and RNA-Seq data. *Bioinformatics* 29(17)**,** 2153-2161. doi: 10.1093/bioinformatics/btt363.
